# Possibilities to Use Physical Simulations When Studying the Distribution of Residual Stresses in the HAZ of Duplex Steels Welds

**DOI:** 10.3390/ma14226791

**Published:** 2021-11-10

**Authors:** Jaromír Moravec, Šárka Bukovská, Martin Švec, Jiří Sobotka

**Affiliations:** Department of Engineering Technology, Faculty of Mechanical Engineering, Technical University of Liberec, 46117 Liberec, Czech Republic; sarka.bukovska@tul.cz (Š.B.); martin.svec@tul.cz (M.Š.); jiri.sobotka@tul.cz (J.S.)

**Keywords:** duplex stainless steel, residual stresses, X2CrMnNiN21-5-1 steel, X-ray diffraction analysis, physical simulations, Gleeble 3500

## Abstract

Dual phase steels combine very good corrosion resistance with relatively high values of mechanical properties. In addition, they can maintain good plastic properties and toughness at both room temperature and lower temperatures as well. Despite all the advantages mentioned above, their utility properties can be reduced by technological processing, especially by the application of the temperature cycles. As a result, in the material remain residual stresses with local stress peaks, which are quite problematic especially during cyclic loading. Moreover, determining the level and especially the distribution of such residual stresses is very difficult for duplex steels both due to the structure duality and in light of the very small width of the heat-affected zone (HAZ). This is why the paper presents the possibilities of using physical simulations to study the effect of temperature cycles in residual stresses’ magnitude and distribution, where it is possible to study the HAZ in more detail as well as on a much larger sample width due to the utilization of special samples. In the thermal–mechanical simulator Gleeble 3500, temperature-stress cycles were applied to testing samples, generating stress fields with local peaks in the testing samples. In addition, the supplied steel X2CrMnNiN21-5-1 had different phase rations in the individual directions. Therefore, as the residual stresses were measured in several directions and at the same time, it was possible to safely confirm the suitability of the used measurement method. Moreover, the effect of the stress and strain on the change of partial phases’ ratios was observed. It has been experimentally confirmed that annealing temperatures of at least 700 °C are required to eliminate local stress peaks after welding. However, an annealing temperature of 550 °C seems to be optimal to maintain sufficient mechanical properties.

## 1. Introduction

Austenitic–ferritic duplex stainless steels (DSS) are very often used in technical practice due to their properties such as sufficient corrosion resistance, very good mechanical properties, and guaranteed weldability [1,2]. The content of austenite and ferrite promoting elements must be balanced in order to achieve the required ratio of the two phases in the structure. However, even a very small change in chemical composition can cause a significant change in the structure and properties of steel. In addition to that, the ferrite–austenite ratio depends not only on the chemical composition of steel but also on the method of its processing [3,4,5].

Compared to conventional structural steels, fully austenitic and duplex steels have lower values of the thermal conductivity coefficient and higher values of the linear thermal expansion coefficient. Therefore, it is necessary to take into account large deformation during their welding. To eliminate deformation, duplex steels are usually welded with a sufficiently rigid clamping. Nevertheless, as a result, in the weld area, residual stresses with significant peaks are determined [2,6,7,8]. And right these stress peaks reduce the fatigue life of welded parts under cyclic loading.

Another characteristic that occurs during the welding of duplex steels is a change in the mutual ratio of ferrite and austenite depending on the welding conditions. The heat input value has an especially significant influence, alongside the maximum achieved temperature and used cooling rate [2,3,4,5,9,10]. From works dealing with this issue, it was revealed that when increasing the heat input value and temperature, there is a higher ratio of austenite in the duplex structure [2,3]. Conversely, the higher the cooling rate, the higher the ratio of the ferritic phase [4,5,9]. It is well-known that the phase ratio is absolutely essential to determine the resulting residual stresses by using diffraction analyses [1,2,11,12]. The phase ratio also has a partial effect on the mechanical properties and corrosion resistance [2,5,13]. It is possible to eliminate such influence of the changing phase ratio when measuring residual stresses by using destructive methods such as, e.g., the hole-drilling method [8,14]. However, as a disadvantage, the destruction of the testing sample in the measuring point occurs and thus there is no possibility to use it for further testing.

During welding, but also under static [15] or cyclic [16] loading of DSS, macroscopic and microscopic residual stresses occur. Macroscopic residual stresses (type I) occur in areas with a size of a few millimeters or even larger and are usually caused by the used technological processing or by direct mechanical loading. Microscopic residual stresses are phase specific and can be further divided into type II (grain level stress) and type III (crystal lattice level) stresses [12]. In the case of duplex steel, stress of type II can occur because of the properties of mismatch among different phases, which include elastic mismatch, thermal misfit, and plastic misfit [17,18]. In general, it can be stated that the measurement of the residual stresses after welding is truly demanding for duplex steels but it is already a mastered measurement methodology. Residual stresses on the surface of DSS can be measured by using the XRD (X-ray diffraction) method [1,2,11,15,16,19,20] or via the use of the strain gauge method with the layering technique [7]. Residual stresses under the material surface can be then measured both destructively by, e.g., the hole-drilling method [8,14] and non-destructively by the neutron diffraction [12].

However, the present work did not aim to study the individual types of residual stresses but rather an effort was made to present the possibility of using welding process physical simulations to perform a detailed study of the residual stresses distribution in the HAZ as well as the possibility to influence them by stress relief annealing. Therefore, the residual stresses are, in this paper, evaluated comprehensively using the XRD method in partial areas of the HAZ. This is why the essence of such XRD measurement methods are briefly presented below.

X-ray diffraction is based on the scattering of X-rays on the crystals of a material. Based on the X-rays, scattering measures changes in the lattice interplanar spacing (generally d-spacing), which are caused by the applied stress. These changes result in the change of Bragg angle θ (reflection of X-rays). Determined deformations are then recalculated by using equations from the theory of elasticity. Radiation scattering on the adjacent planes leads to an interference maximum in the direction of angle θ if the Bragg′s law (1) is satisfied [1,21,22,23].

(1) $$n\cdot \lambda =2\cdot d_{\mathrm{hkl}}\cdot \sin\theta$$

where n—order of diffraction, λ—wavelength of the incident radiation, d_hkl_—interplanar spacing of the adjacent lattice planes, and θ—Bragg angle.

**Figure 1:**
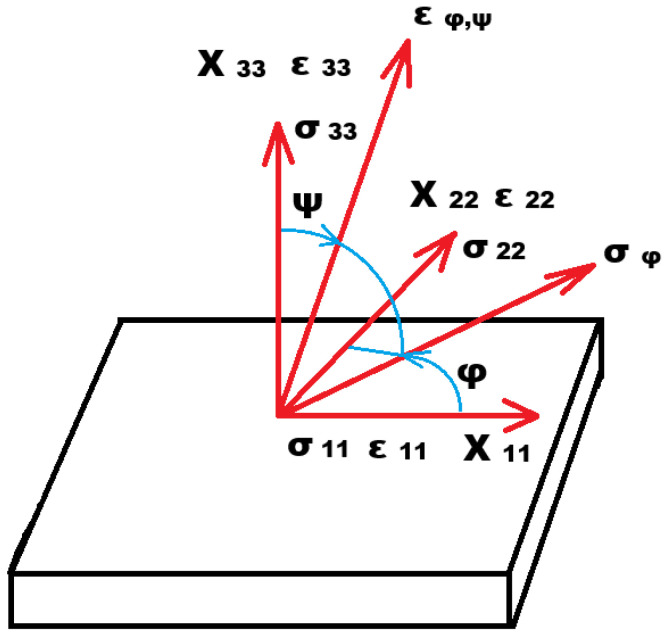
Schematic illustration of the individual stress and strain components on the material surface [21].

By substituting the values of the interplanar spacing without stress and in the deformed state, Equation (2) for the lattice deformation is obtained.

(2) $$\varepsilon =\frac{d-d_{0}}{d_{0}}=-\cot\theta_{0}\left(\theta -\theta_{0}\right)$$

where d—interplanar spacing in the deformed state, d_0_—interplanar spacing in the undeformed state, θ_0_—Bragg angle of the crystal without stress, and θ−θ_0_—angle offset of the interference maximum. Deformation can be applied in the general direction when every direction of the deformation can be defined using angles φ and ψ, which determine the lattice deformation ε_φψ_ expressed by Equation (3). By identifying the lattice deformation ε with the deformation ε_φψ_, the basic equation of the stress measurement according to the so-called method $\sin^{2}\psi$ is obtained.

(3) $$\varepsilon_{\varphi \psi }=-\cot\theta_{0}\left(\theta -\theta_{0}\right)=\frac{\nu +1}{E}\sigma_{\varphi }\sin^{2}\psi +\frac{-\nu }{E}\left(\sigma_{11}+\sigma_{22}\right)$$

where E and v—elastic constants, φ and ψ—azimuthal and polar angles in the spherical coordinate system, σ_ii_—stress in the given direction, and σ_φ_—stress in the direction of angle φ on the plane of the material surface. The individual components of the stress and strain on the material surface are schematically shown in Figure 1.

From the Bragg’s law and from Equation (3) expressing ε_φψ_ follows Equation (4) for the calculation of the stress components σ_φ_.

(4) $$\sigma_{\varphi }=\frac{E}{\nu +1}\cot\theta_{0}\frac{\partial \theta_{\varphi \psi }}{\partial \sin^{2}\psi }$$

Using X-ray diffraction, it is possible to apply the NDT (Non-destructive testing) method to measure the surface stress of the tested material in a relatively small area usually within the range from 0.2 to 0.8 mm^2^. However, despite such a small measured area, residual stresses in the HAZ of the welds can be determined only with limited sensitivity. The reason for this concerns the very small width of the HAZ, which does not exceed 1.5–2 mm for a majority of the fusion welding methods. Due to the focusing of the beam, the individual areas overlap with each other and are thus also partially averaging. As will be shown below, this disadvantage can be eliminated by using special testing samples and the physical simulations of the temperature-stress cycles performed by using the thermal–mechanical simulators. In this way, the magnitude of the HAZ can be more than five times higher.

## 2. Materials and Methods

Steel marked as X2CrMnNiN21-5-1 according to standard ISO 10027-1 and 1.4162 according to standard ISO 10027-2 was used for the experiments. It is a duplex steel classified according to its resistance against pitting corrosion into the lean duplex group with a molybdenum content (pitting resistance equivalent number PREN = 26.4). The steel used for the experiments was supplied in the form of rolled plates with the dimensions of 500 × 600 × 10.5 mm^3^. Its chemical composition was determined by using a Bruker Q4 Tasmann spectrometer (Karlsruhe, Germany). The measured chemical composition of the tested steel, including the composition of such steel as defined by the standard EN 10088-1, is given in Table 1.

The mechanical properties YS (Yield Strength), UTS (Ultimate Tensile Strength), A_g_, and A_30_ were measured at room temperature on the supplied basic material as well as on samples annealed at the following temperatures: 550, 650, and 700 °C, each for 2 h. The mechanical properties of the basic material were determined both in the rolling direction and in the direction perpendicular to the rolling direction. In the case of the annealed samples, mechanical properties were determined just in the direction perpendicular to the rolling direction. The static tensile test was performed on testing machine TIRA Test 2300 (TIRA GmbH, Schalkau, Germany) according to the standard EN ISO 6892-1.

The real temperature cycles and boundary conditions necessary to perform physical simulations on the Gleeble 3500 device (Dynamic System Inc., New York, NY, USA) were obtained from a real welding experiment carried out by the TIG method. A Migatronic Navigator 3000 (MIGATRONIC A/S, Fjerritslev, Denmark) welding power supply with a machine torch attached to a linear automat was used for the welding. Welding parameters were as follows: the current was 150 A and travel speed was 0.2 m·min^−1^. Process parameters were scanned by using a WeldMonitor system (DIGITAL ELECTRIC, Brno, Czech Republic) with a data scanning frequency of 25 kHz and temperature cycles were measured with a DiagWeld (Technical University of Liberec, Liberec, Czech Republic) apparatus with a data scanning frequency of 50 Hz.

**Figure 2:**
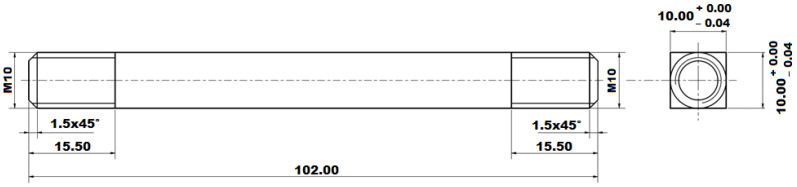
Drawing of the sample used for the physical simulation with subsequent measurements of the residual stresses by XRD.

**Figure 3:**
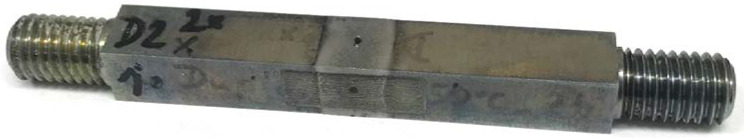
Example of the sample used for the physical simulation of the welding process (the figure shows a sample after the application of the temperature cycle of 1386 °C).

For physical simulations and for the subsequent measurement of residual stresses, specially shaped specimens were designed; see Figure 2. The reason for the shapes and dimensions of the samples concerns both the possibility to clamp it without any problems at the thermal–mechanical simulator Gleeble 3500 and allow for the application of temperature cycles corresponding to the real welding. The length of such a sample that is clamped between the high temperature grips is designed so that it is possible to apply temperature cycles in a width of about 6.5 times larger than for the HAZ in real welding. At the same time, the length of the sample must not be too long because of the ability to achieve a cooling rate corresponding to the real welding cycles. Due to the threads at the ends of the sample, it is possible to fix the sample, thus there is no relative movement during the occurrence of tensile and compressive stresses, and the boundary conditions of the clamping can be defined, which correspond to the reality. A square cross-section was selected concerning the measurement of residual stresses by the XRD method. In the case of samples with a circular cross-section, it is necessary to compensate the Bragg angle θ caused by the radius of the sample. Therefore, a square cross-section sample was designed, which has a sufficiently large planar area for measuring residual stresses and, at the same time, also has symmetry for the application of thermal stress cycles on the device Gleeble 3500. Figure 3 shows a real sample, in this case, after the application of the temperature cycle of 1386 °C. In the center of the sample, the area affected by the high temperatures and deformation achieved during the cycle is quite apparent.

**Figure 4:**
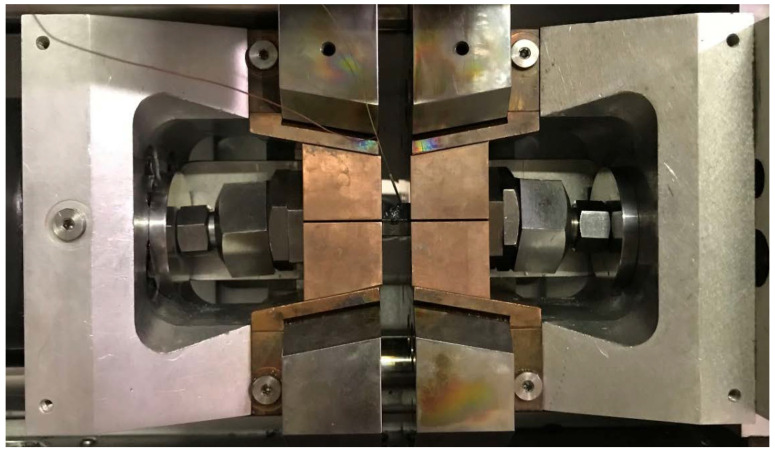
Clamping of the square cross-section sample in the Gleeble 3500 device.

The thermal–mechanical simulator Gleeble 3500 allows for achieving a heating rate of up to 10,000 °C·s^−1^, which is completely sufficient for the simulation of the welding processes. However, the problem concerns achieving the correct temperature distribution in the sample and to ensure a sufficiently high cooling rate. To avoid these specifics, a combination of the proper type of high temperature grips and the so-called free length of the sample between the high temperature grips can be used. For our type of experiments, copper high-temperature grips with a solid cross-section and a free length of the sample between the grips of 10 mm were used. The own clamping of sample in the Gleeble device is shown in Figure 4. In the middle of the testing sample, a control thermocouple was welded to control the temperature cycle via feedback. For elimination of the sample movement, we used distance bold U-Jacks. The boundary conditions defining the clamping stiffness were realized by choosing the appropriate temperature-stress cycle control method. In this case, a deformation control was used, thus the sample could not expand freely and was plastically deformed after exceeding the yield strength at a given temperature.

The X-ray device PROTO iXRD COMBO (Proto Manufacturing Inc., Ontario, Canada) was used to measure the residual stresses. Since the tested material was duplex steel, the residual stress of ferrite and the residual stress of austenite had to be measured separately in each region. The final value of the total residual stress for each measured point was then determined by using the weighted arithmetic mean according to the relevant phase ratio. Ferritic areas were measured with a chromium X-ray (voltage of 25 kV, current of 4 mA, and wavelength of Kα = 2.291 A) and austenitic areas with a manganese X-ray (voltage of 20 kV, current of 4 mA, and wavelength of Kα = 2.103 A). The actual measurement of the residual stresses was performed in the transverse direction from the center of the testing sample to the sides, up to a distance of 16 mm (step 1 mm, i.e., 33 measured points).

The percentage distribution of austenite/ferrite phases and the grain size of the individual phases were determined by the EBSD (Electron Back Scatter Diffraction) method carried out on the scanning electron microscope (SEM) Tescan Mira 3 (Tescan Orsay holding a.s., Brno, Czech Republic). For this, EBSD analysis was used with an Oxford symmetry detector (Oxford Instruments, High Wycombe, UK) with the following process parameters: high voltage of HV = 15 kV, step size of 0.7 µm, and a scanned area with dimensions of 1500 × 1500 µm^2^.

The heat treatment of the samples before applying the temperature-stress cycle was performed in the vacuum furnace Reetz (HTM Reetz GmbH, Berlin, Germany) at 550 °C for 2 h. The heating rate of the sample was 1.5 °C·min^−1^ to a temperature of 250 °C and 4 °C·min^−1^ for the temperature range 250 up to 550 °C (700 °C). The cooling rate of the sample was then 5 °C·min^−1^. During the heat treatment, the vacuum in the furnace was 7 × 10^−5^ mbar. Under the same conditions, stress-relief annealing was performed after the application of temperature-stress cycles. This was carried out at temperatures of 450, 500, 550, 600, 650, and 700 °C, each with a soaking time of 2 h.

## 3. Experiment

### 3.1. Mechanical Properties of the Material

Measured mechanical values of the basic material are given in Table 2. Samples taken in the rolling direction are designated as RD-Tx (rolling direction—annealing temperature) and samples taken in a direction perpendicular to the rolling direction are designated as PD-Tx (perpendicular direction—annealing temperature), where Tx indicates the temperature at which the sample was annealed. Samples marked as T23 were not annealed and correspond to the base material taken in the relevant directions. From the results, it is clear that such directions have very little effect on the material mechanical properties. The difference in the average values of the yield strength YS and the ultimate tensile strength UTS was lower than 3% and did not exceed 5% for ductility. Therefore, only samples taken in a direction perpendicular to the rolling direction were used at higher temperatures. From Table 2, it can be seen that the higher the annealing temperature, the lower the yield strength YS. It decreased by 25% and 30% compared to the basic material. The ultimate tensile strength UTS then decreased by only 9% and 11%. On the contrary, there was a significant increase by 35% and 71% in the case of homogeneous ductility.

### 3.2. Determination of the Phases and Grain Size Ratios

**Figure 5:**
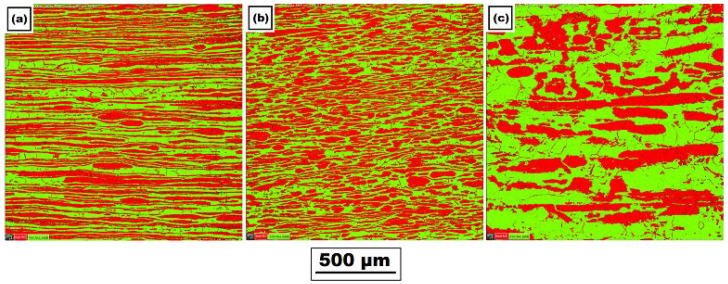
Distribution of ferrite (green) and austenite (red) in the basic material along with the following directions: (**a**) *x*-axis (direction perpendicular to the rolling direction), (**b**) *y*-axis (rolling direction), and (**c**) *z*-axis (material thickness direction).

Figure 5 shows the mutual ratio of the austenite (red color)/ferrite (green color) phases in the basic material in three mutually perpendicular directions obtained by EBSD analysis. The individual directions correspond to the rolling direction (*y*-axis), the direction perpendicular to the rolling direction (*x*-axis), and the section in the material thickness direction (*z*-axis). The specific values of austenite and ferrite content in the base material are summarized in Table 3.

**Figure 6:**
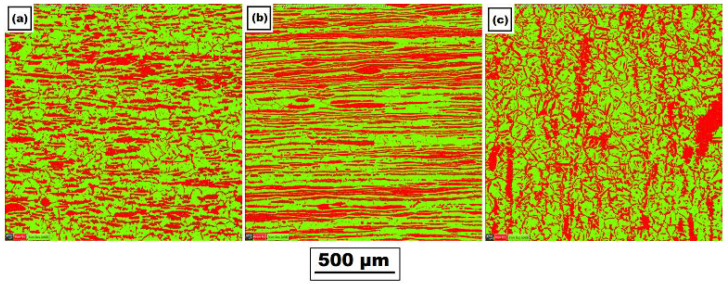
Distribution of ferrite (green) and austenite (red) in the area achieving the maximal temperature of 1386 °C along with the following directions: (**a**) *x*-axis (direction perpendicular to the rolling direction), (**b**) *y*-axis (rolling direction), and (**c**) *z*-axis (material thickness direction).

To verify the effect of the applied temperature on the austenite/ferrite phase ratio, the EBSD phase analysis was also performed in the material area, where a maximum temperature of 1386 °C was reached during testing. The resulting phase ratios are shown in Figure 6 and specific phase ratio values are given in Table 3.

**Figure 7:**
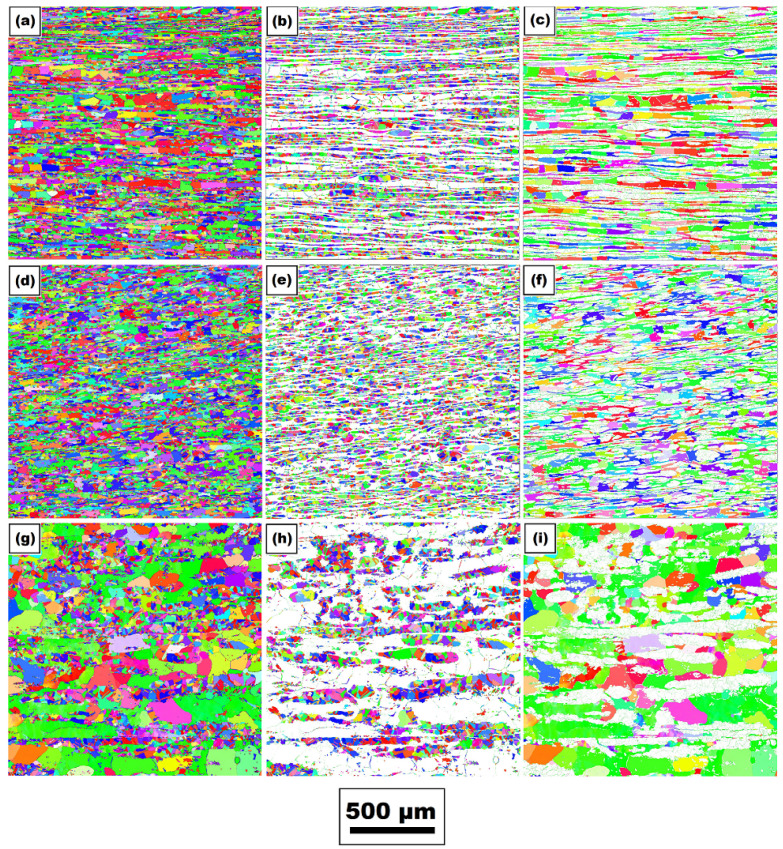
Illustration of grains in the basic state: in *x*-axis (PD)—(**a**) overall image of grains; (**b**) austenite grain; and (**c**) ferrite grain; in *y*-axis (PD)—(**d**) overall image of grains; (**e**) austenite grain; and (**f**) ferrite grain; in *z*-axis (PD)—(**g**) overall image of grains; (**h**) austenite grain; and (**i**) ferrite grain.

**Figure 8:**
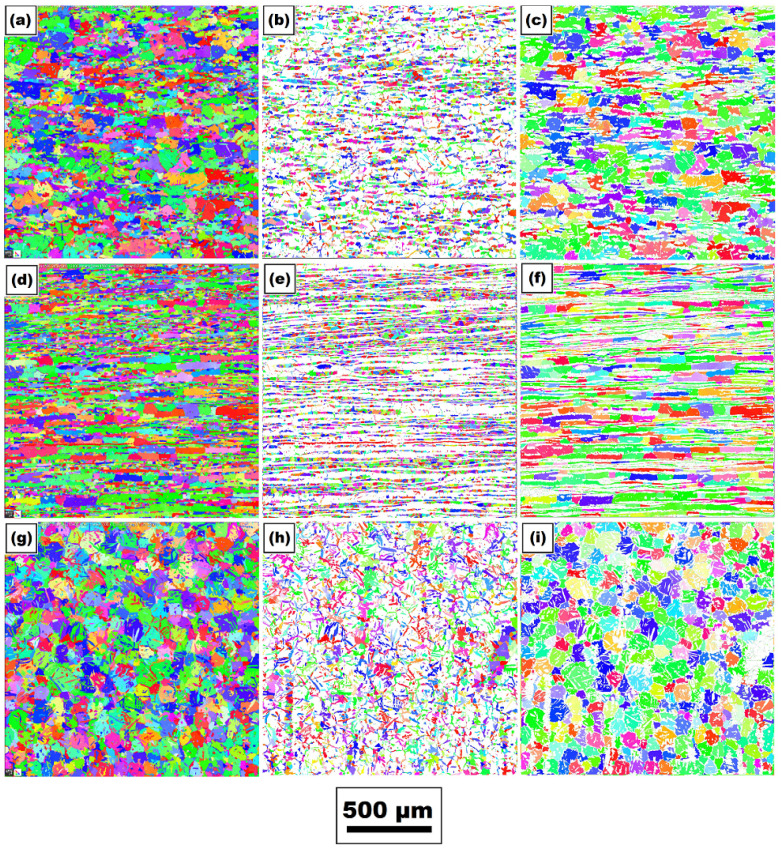
Illustration of grains in the area achieving the maximal temperature of 1386 °C: in *x*-axis (PD)—(**a**) overall image of grains; (**b**) austenite grain; and (**c**) ferrite grain; in *y*-axis (PD)—(**d**) overall image of grains; (**e**) austenite grain; and (**f**) ferrite grain; in *z*-axis (PD)—(**g**) overall image of grains; (**h**) austenite grain; and (**i**) ferrite grain.

An analysis of the grain size of the individual phases in all three directions was also performed both for the basic material and for the material, where the physical simulation of the welding cycle was conducted. In the sample after the physical simulation, the grain size was analyzed in the area of maximum temperatures, which varied from about 1280 °C to 1386 °C, with the maximum temperature in the middle of analyzed area. An illustration of the grains as well as the distribution of the grains regarding the individual phases (austenite and ferrite grains) for both states of the tested material is shown in Figure 7 and Figure 8. Specific values of individual phases’ grain sizes for both states of the material are written in Table 4.

### 3.3. Measurement of the Temperature Cycles and Determination of the Boundary Conditions for Physical Simulations

**Figure 9:**
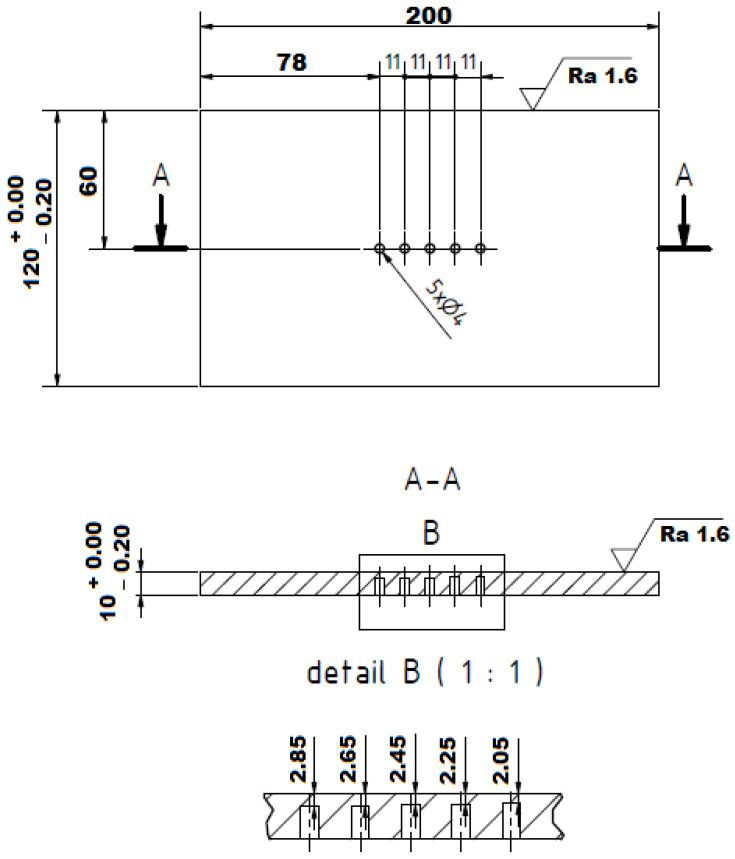
Drawing of the testing plate (setting the real welding conditions).

The physical simulations of the processes taking place in the HAZ of the welds must be based on the real welding conditions. Thus, this concerned the course of the temperature cycles close to the fusion line and the control temperature cycle, assuming the lower limit of the HAZ, i.e., the cycle with a maximum temperature of about 700 °C. Obtaining the temperature cycles from the fusion line is very difficult in real welding only because of the accuracy of the location of the thermocouple close to the expected weld. Therefore, a special welding experiment was prepared. In the first phase the experiment, a run was performed on the plate surface and, at the same time, the geometry of such a weld as well as the penetration stability in the welding direction were evaluated. Based on this geometrical information, a special plate was prepared. It had five holes with a diameter of 4 mm milled from the lower part and with a depth graduated by 0.2 mm from the assumed depth of the weld penetration. Thus, the bottom of the first hole was 0.1 mm away from the assumed weld penetration and this distance was gradually increased by 0.2 mm at each hole. The drawing of the special test plate is shown in Figure 9. S-type thermocouples were joined by CD welding to the bottom of individual holes and onto the surface of this plate achieved by welding via the TIG method. The following process parameter effective values were measured by the WeldMonitor system: current I = 153.4 A; voltage U = 21.8 V; and travel speed v_s_ = 0.201 m·min^−1^. The total heat input value was Q = 9.98 kJ·cm^−1^.

**Figure 10:**
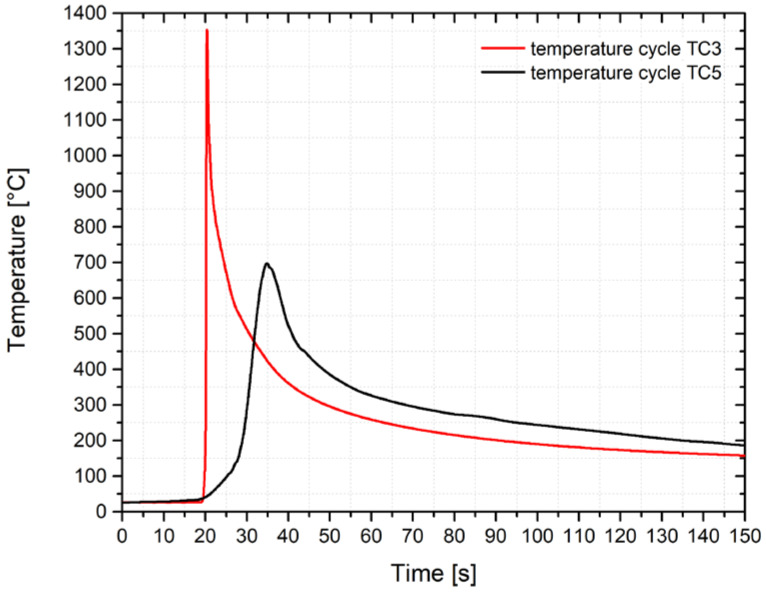
Temperature cycles measured by thermocouples TC3 and TC5.

Thermocouples located in the first two holes were overflowed with a weld pool and measured a maximum temperature of 1587 °C and 1521 °C. A reason for this concerns the fact that there was probably a deterioration in the plate compactness and thus in the local change of the conditions for heat dissipation. Thermocouple TC3 in the third hole recorded a temperature cycle with a maximum temperature of 1386 °C and after metallographic evaluation, a distance of 0.09 mm from the bottom of the hole with the thermocouple to the fusion line was measured. This temperature cycle could be used with sufficient accuracy for the physical simulations of the processes in the HAZ. The fifth thermocouple TC5 then measured a temperature cycle with a maximum temperature of 692 °C and with a determined distance from the fusion zone of 0.88 mm. The measured courses of the temperature cycles are shown in Figure 10, while there is an offset of the time axis to ensure that both cycles start at the same time.

### 3.4. Physical Simulation of the Processes in the HAZ of the Welds from X2CrMnNiN21-5-1 Steel

**Figure 11:**
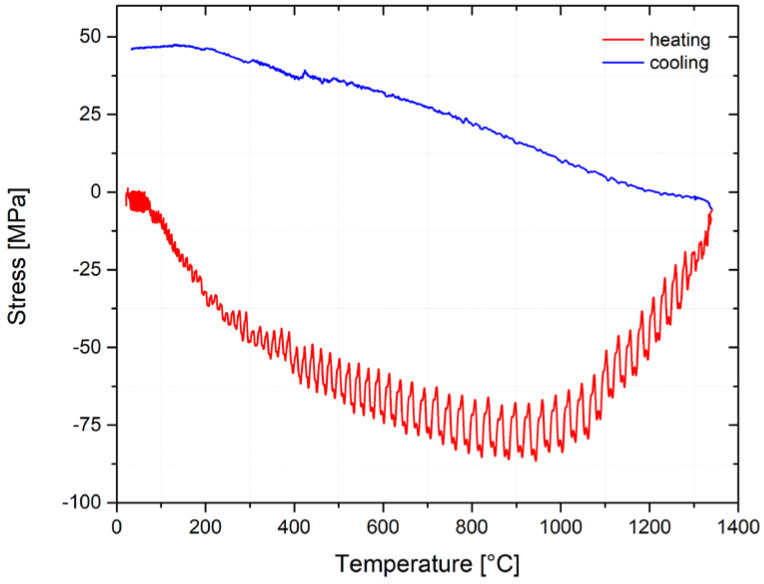
Stress vs. temperature in the sample; temperature cycle of 1386 °C.

The thermo–mechanical simulator 3500 was used for the physical simulations of the processes in the HAZ of the welds. The simulations used copper high-temperature grips with a full cross-section and a free sample length of 10 mm. The temperature range of 1386–692 °C, which was reached in a real experiment at a distance of about 0.8 mm, was, in this case, possible to be newly measured on a sample at a distance of about 5.3 mm, symmetrically distributed from the center of the sample. The whole temperature-stress cycle was controlled by means of an extensometer, on which the condition of zero movement of the sample was set. Our own record of the stress course in the sample, where the temperature cycle TC3 was applied, is shown in Figure 11. The red part of the measured curve shows the heating part of the temperature cycle and the blue part of this curve represents the stress resulting in the sample during its cooling. From the course of the stress, it is clear that increasing temperature also increases the compressive stress. This is valid until the compression yield stress is reached at a given temperature (approximately at 250 °C). Subsequently, the sample is plastically deformed. At the temperature of about 900 °C, there is a significant decrease of compressive stresses due to an intensive decrease of the yield strength at a given temperature. After reaching the maximum cycle temperature, the sample cools down and, instead of compressive stresses, there are tensile stresses. These stresses increase gradually with the decreasing temperature. However, this course shows the total value of stresses in the whole testing sample and does not correspond with the residual stresses in the individual parts of the sample.

### 3.5. Measurement of the Residual Stresses

**Figure 12:**
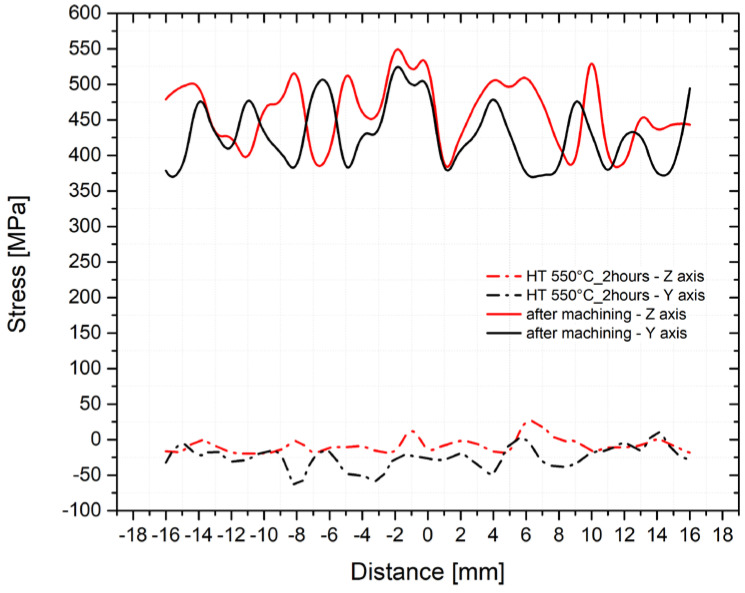
Residual stresses after machining and after HT (*y*-axis—black and *z*-axis—red).

Considering that significantly different ratios of phases were determined for the basic material in the material thickness direction (*z*-axis), residual stress measurements were performed before applying temperature cycles to the sample separately for the *y* and *z*-axis. The resulting values of the residual stresses for both these directions are shown in Figure 12. The distance indicated on the *x*-axis was measured from the center of the sample (see Figure 3). From the results, it is obvious that the machining-left significant tensile stresses in the surface layer have values varying from 400 to 500 MPa. The magnitude of the residual stresses is similar for both directions despite the significantly different mutual ratios of ferrite and austenite. However, to compare the effects of the individual temperature-stress cycles, it is appropriate to start from a stress level close to zero. Therefore, the machined samples were annealed in a Reetz vacuum furnace at 550 °C for 2 h and re-measured in the same way and at identical points as in the previous case. Results for both directions are also included in Figure 12. At the same time, through EBSD analysis, we also tested whether the annealing changed the phase ratio in the individual evaluated directions. The determined deviations were minimal. From a comparison of the curves in Figure 12, it is clear that the annealing temperature is sufficient to remove the stress in the surface layer after machining.

**Figure 13:**
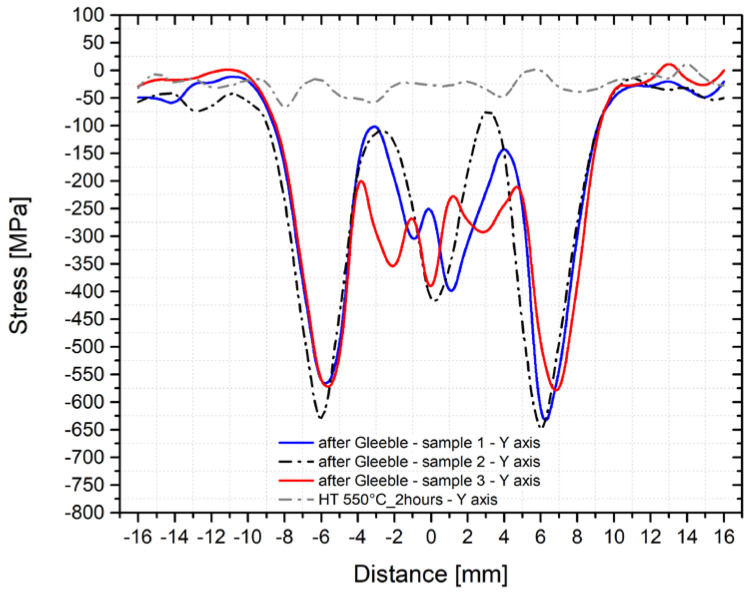
Course of the transverse residual stresses along the *y*-axis after the application of the temperature cycle (three samples annealed at a temperature of 550 °C for 2 h).

**Figure 14:**
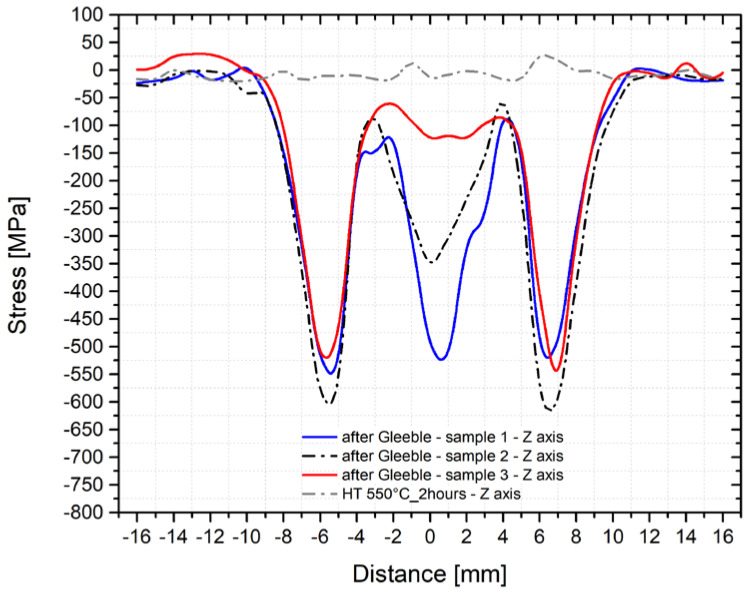
Course of the transverse residual stresses along the *z*-axis after the application of the temperature cycle (three samples annealed at a temperature of 550 °C for 2 h).

To assess the repeatability of such physical simulations and the XRD measurement methodology, an identical temperature cycle was applied also in the thermo-mechanical simulator Gleeble 3500. Here, we used three samples annealed at 550 °C for 2 h. Concerning the different phase ratios, the subsequent measurement of the residual stresses was performed along with the *y*-axis and *z*-axis. Measured courses of the transverse residual stresses in the *y*-axis after the application of the temperature-stress cycle for all three samples are shown in Figure 13 and in Figure 14 for the *z*-axis as well. The gray line shows the course of stress in the initial state (sample number 2, i.e., after annealing at 550 °C for 2 h).

**Figure 15:**
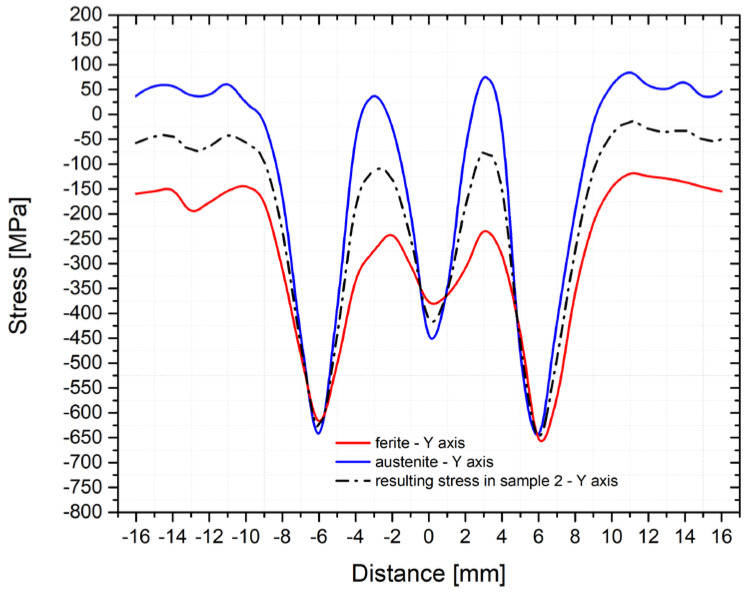
Course of the residual stresses for individual phases (A and F) and the resulting stress in sample number 2 (*y*-axis direction).

**Figure 16:**
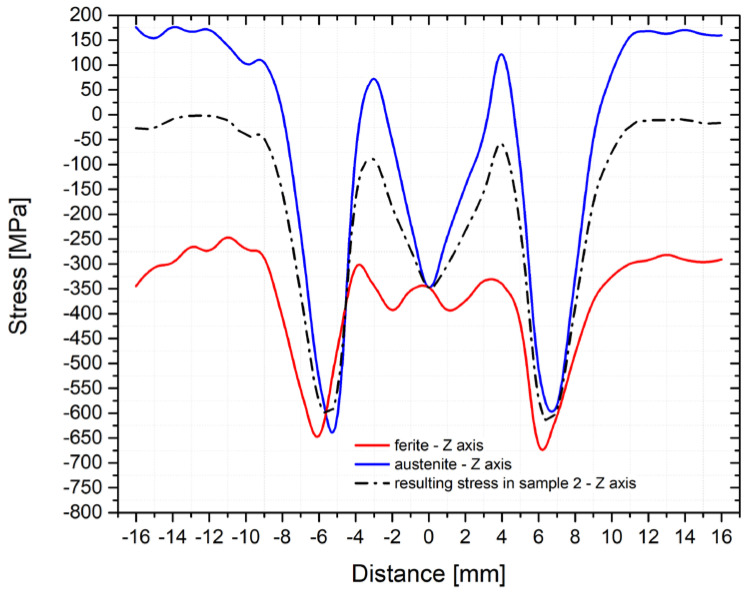
Course of the residual stresses for individual phases (A and F) and the resulting stress in sample number 2 (*z*-axis direction).

As can be seen from the figures above, the residual stress curves were almost identical for all three samples. Only in the area of the achieved maximum temperature (where the largest plastic deformation of the testing sample was located) were more significant differences in the direction of the *z*-axis found. To give an overview of how the individual structural phases contributed to the residual stresses, Figure 15 and Figure 16 were made. In Figure 15, courses of the transverse residual stresses in austenite and ferrite along the *y*-axis (in Figure 16 along the *z*-axis) are shown. In both cases, these stresses were measured on sample number 2.

**Figure 17:**
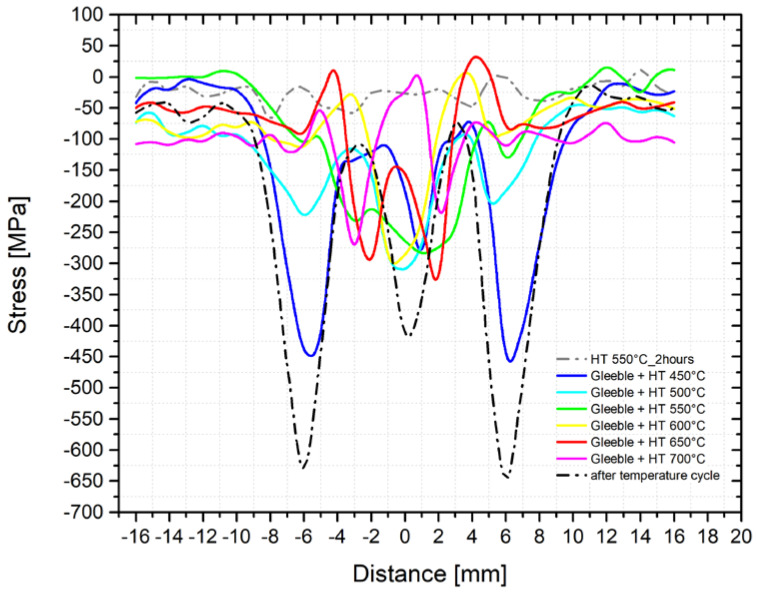
Course of the residual stresses after the application of the stress-relief annealing at different temperatures (*y*-axis direction).

**Figure 18:**
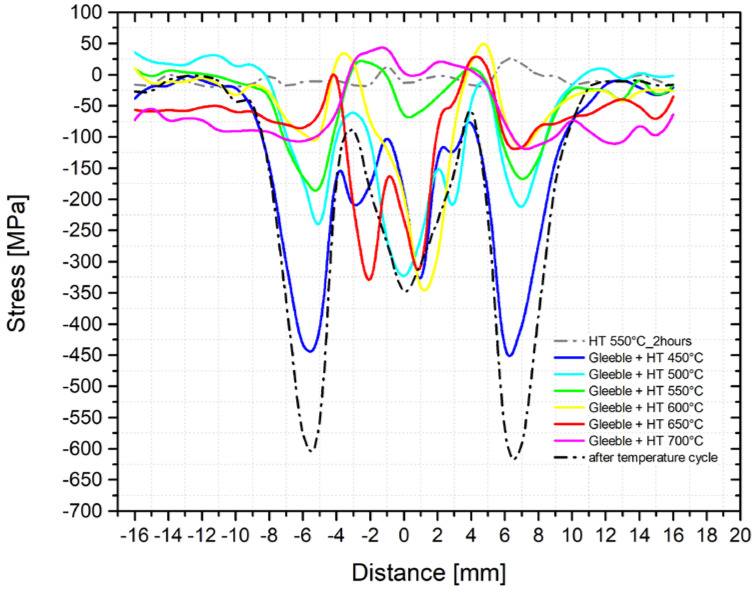
Course of the residual stresses after the application of the stress-relief annealing at different temperatures (*z*-axis direction).

Residual stress and especially local stress peaks have a negative effect on the component′s lifetime. Therefore, techniques to reduce residual stresses are applied, especially after welding. The most common techniques used include methods of reducing residual stresses. To study the proper magnitude of annealing temperatures, we used samples subjected to the temperature-stress cycle in the simulator Gleeble 3500. Moreover, before the thermal treatment, we measured residual stresses along with the individual directions and these courses are shown in Figure 13 and Figure 14. The samples prepared in this way were subsequently placed in the vacuum furnace Reetz and subjected to stress-relief annealing at the following temperatures: 450, 500, 550, 600, 650, and 700 °C, always for 2 h. The determined residual stress distribution after the application of these individual types of heat treatments is shown in Figure 17 (*y*-axis direction) and Figure 18 (*z*-axis direction). It is clear from the stress profile that at the annealing temperature of 450 °C, the maximum stress peak reduced by about 150 MPa was found. Moreover, the situation after annealing at 650 °C is much more interesting because not only a reduction of the residual stresses’ magnitude was observed but also their redistribution. Thus, stress peaks both decreased and shifted closer to the fusion line, i.e., to the area with the maximum temperature.

## 4. Discussion

The present study in this paper shows a new way to study the residual stresses in the HAZ of welds. Generally, with the help of physical simulations carried out in thermal–mechanical simulators, it is possible to create conditions corresponding to real welding. Moreover, there is a possibility to create these conditions repeatedly, which can be quite problematic in the real welding process. Another advantage concerns the possibility of studying changes over a much larger area, thus with higher accuracy. As shown in Section 3.3. regarding measuring temperature cycles, the temperature difference from 1386 °C to 692 °C was achieved only at 0.8 mm. This means that even when using a 0.5 mm filter to focus the X-ray, there exists the possibility to perform only two measurements in a given area with minimal mutual overlap. In addition, by using physical simulations and special testing samples, there exists the possibility to extend the same area up to a length of 5.2 mm, i.e., a 6.5 times-larger area. In addition, it is already possible to place five to six measurements with zero overlap in this area. Furthermore, there is also a possibility to study structural changes; in this case, the mutual ratio of duplex phases and grain size. The higher the area for evaluation by EBSD analysis, the lower the influence of inhomogeneities and structural anomalies. As the resulting residual stress in the measured area is calculated as a weighted ratio of the individual structural components’ (ferrite and austenite) stresses, there is a lower influence of inhomogeneities and structural anomalies on the measured values of residual stresses.

Due to the new design of the testing samples and also due to the measurement of temperature cycles in the HAZ during welding, as carried out on steel X2CrMnNiN21-5-1, it was possible to determine the boundary conditions to create the physical simulations. These were subsequently used to simulate welding processes, which take place in the HAZ during the welding of the tightly clamped X2CrMnNiN21-5-1 steel parts, namely at the heat input value of Q = 9.93 kJ·cm^−1^. The first and very fundamental finding regarding the supplied steel X2CrMnNiN21-5-1 was that the mutual phase ratio differs in the individual directions. In the rolling direction and perpendicular to the rolling direction, the same values of the ferrite and austenite ratios were measured, respectively, but in the material thickness direction, this ratio was significantly different. It was not possible to confirm whether any other authors solved a similar problem in the relevant literature. This may be because the residual stresses in the works cited in this paper were measured only on the surface of real welds for the XRD method [1,2,6], the strain gauge method with the layering technique [7], or, in this case, the hole drilling method [8,14]. In light of the neutron diffraction, the residual stresses were measured only in the thickness direction, i.e., again in one direction [12].

The different phase ratios measured in the different directions are very advantageous to confirm the suitability of the used measurement methodology. Thus, a sample with an identical temperature cycle can be evaluated by measuring partial residual stresses in directions with different phase ratios. The fact that significantly different partial residual stresses were measured in the direction of the *y*-axis (Figure 15) and *z*-axis (Figure 16), while the resulting stresses were almost identical, confirms that the proposed experimental methodology and method of our own measurement and evaluation are correct and relevant.

The profile of the residual stresses distribution corresponds to the presumptions concerning the course of transverse stresses in the welded joint. Generally, in the case of structural steel welded joints, significant compressive stresses’ peaks at greater distances from the fusion line outside the HAZ are noted. These stresses are changed to tensile ones in the HAZ, while there is a significant decrease of tensile stresses in the area of maximum temperatures [20,24,25,26]. Exactly such a shape of the residual stresses was determined also during the physical simulations of welding steel X2CrMnNiN21-5-1. However, all detected residual stresses were compressive ones. A similar result was obtained by, e.g., Ouali et al. [2]. In light of the residual stresses course in the individual phases, it is clear that the ferritic phase revealed only compressive stresses, while in austenite, predominately tensile stresses were revealed. The reason for this is, on the one hand, because of different volume expansion coefficients, but, on the other hand, it is mainly due to the composite structure of the material, where the individual areas influence each other. As another important factor, there are different mechanical properties of such partial phases, as well as a relatively high temperature stability of ferrite in the duplex structure. In addition, when the temperature-stress cycle is applied, the stress accumulates non-uniformly. Moreover, it is affected and redistributed by plastic deformation. Austenitic areas plastically deform earlier, which leads to tensile stresses [16]. In the ferritic phase, compressive stresses occur, which prevent further plastic deformation [16]. This is also related to the change in magnitude and to the redistribution of residual stresses after the application of the annealing treatment to reduce this kind of stress. With increasing temperatures, yield strength decreases and thus both the residual stresses caused by plastic micro-deformation decreases and the relaxation process occurs. Furthermore, there is also the recovery of the strain-hardened material, which allows for the transfer of stress peaks to these recovered areas, i.e., closer to the sample center. This presumption is also confirmed by the results of the mechanical tests after annealing at temperatures of 550 and 650 °C when restoration mechanisms took place. Thus, as a result, there was a reduction of the yield strength and an increase of the uniform ductility. In general, there is no standard definition of the optimal annealing temperature to reduce the residual stresses. In principle, a compromise is chosen between the expected magnitude of the residual stresses and the loading of a given part. From the authors′ point of view, a temperature of 550°C appears to be a suitable compromise between the reduction of mechanical properties and the magnitude of residual stresses. The own influence of post-welding heat treatment (PWHT) after the welding of duplex steels was also dealt with in other works, not in terms of the elimination or redistribution of residual stresses, but in light of microstructure [27] and corrosion resistance [24,27].

A very important aspect of the performed experiments also concerns the study of change in the mutual ferrite–austenite ratio. As can be seen from Figure 5 and Table 3 for the basic material, the ferrite–austenite ratio was about 50:50% concerning the unidentified areas in the *x*-axis and *y*-axis directions. In comparison, in the *z*-axis direction, a ferrite–austenite ratio was determined to be 58.6:38.3, with the size of the unidentified area as 3.1%. After the application of the temperature cycle, such ratios in the *z*-axis almost did not change in the area with the maximum temperature. In contrast, in the *x*-axis and *y*-axis direction, austenite content decreased to 38.5 ± 3.5%, thus corresponding to the resulting ratio in the *z*-axis. Therefore, there is a presumption that under the given temperature-stress conditions, there is an equilibrium state.

At the same time, phases were rearranged in a given place in all directions (*x*, *y*, and *z*-axis); see the comparison in Figure 5 and Figure 6. Due to the use of special samples and physical simulations, the HAZ was extended to about a 6.5 times-larger area. Thus, through EBSD analysis, it was possible to detect that such a rearrangement of phases occurred in the temperature range of approximately 1105–1386 °C. In areas with lower temperatures, similar ratios and phase distributions were identified for all directions as in the basic material.

**Figure 19:**
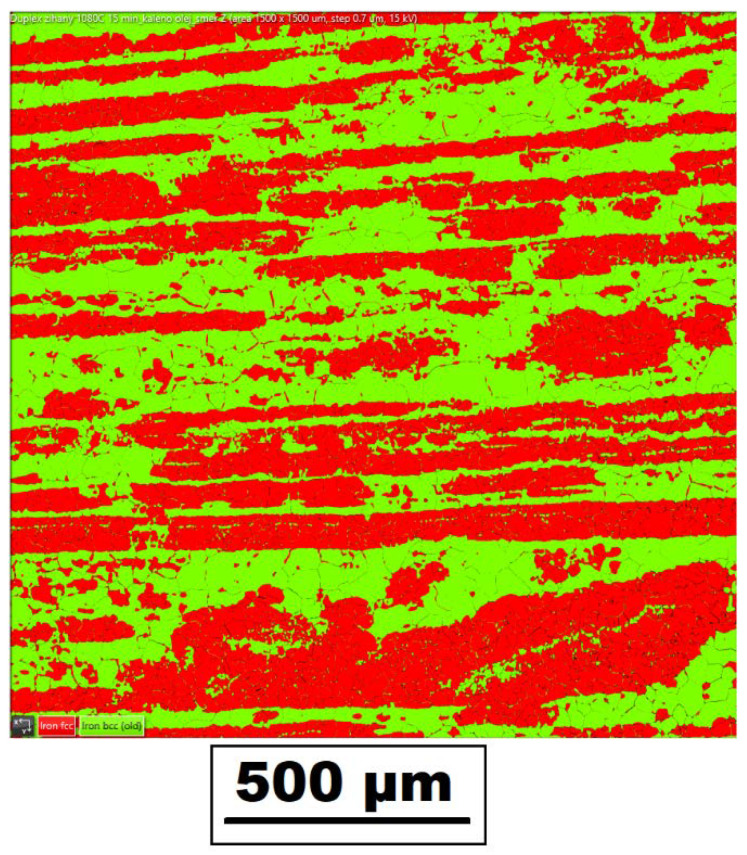
Distribution of ferrite (green) and austenite (red) after annealing at a temperature of 1080 °C with a soaking time of 10 min (t_8/5_ = 5.8 s): ferrite, 47.7%; austenite, 50.1%; and non-identified areas, 2.2%.

The obtained results cannot be fully compared with the available literature because the works published in this area dealt with the influence of temperature and the cooling rate on the change of the ferrite–austenite ratio. The stress-strain factor, which appears to be quite significant for the ratio and distribution of these phases, was not considered here. Studies [2,3] concluded that the maximal austenite ratio in duplex structures is reached after annealing at a temperature range of 1050–1080 °C with subsequent cooling under parameter t_8/5_ for more than 3 s. The above conclusions were also confirmed for the tested steel X2CrMnNiN21-5-1 when used after the heat treatment mentioned above [2,3], wherein the ratio of austenite in the *z*-axis increased from 38.3% to 50.1% (see Figure 19). However, this change was not observed in the temperature range of about 1080 °C on the sample after physical welding simulations. Based on this reality, it follows that the stress-strain state in the material has a significant effect on the transformation of the individual phases’ ratios.

Furthermore, the influence of the used temperature cycle on the grain coarsening in areas with the maximum temperature was also assessed. EBSD analysis revealed that in these areas (maximum temperatures), grain coarsening occurred in both ferritic and austenitic areas. However, the degree of grain coarsening varied in the individual directions and phases (see Table 4).

## 5. Conclusions

In the paper, the possibility to use physical simulations of the welding process to study the residual stresses’ distribution and the behavior of the duplex structure after the application of the temperature cycles was presented. Furthermore, the effect of the stress-relief annealing temperature to reduce residual stresses (especially in the HAZ) was also assessed. The obtained results can be summarized by the following points.

(1) By using physical simulations, it is possible to study the changes occurring in the HAZ of welds over a much larger area and therefore with greater resolution. This can be applied not only to study residual stresses’ distribution and the possibility to influence them but also for the determination of the mutual ratios of duplex phases and grain sizes. The larger the size of an area usable for evaluation, the lower the influence of inhomogeneities and structural anomalies.
(2) When studying the effect of welding on residual stresses performed by diffraction analyses, it is necessary to take into account the possibility of different phase ratios along with the individual directions. Such aspects may be especially important for studies using symmetrical samples regardless of whether they concern physical simulations or changes in structure and residual stresses under static or cyclic loading.
(3) Achieving an almost identical magnitude and distribution mode of residual stresses in two directions with significantly different phase ratios confirms the suitability of the used measurement methods and evaluation as well. Thus, the possibility to use physical simulations and the proposed design of the testing sample is confirmed.
(4) The distribution of transverse residual stresses in X2CrMnNiN21-5-1 steel corresponds to the course of stresses determined at conventional structural steels, but all the resulting stresses are compressive ones. The reason for this arises from the duality and thus the composite behavior of a material.
(5) The utilization of PWHT reduces the maximum value of residual stresses. In addition, at annealing temperatures higher than 550 °C, there is, at the same time, a transfer of peak stresses into the more plastically deformed areas, in this case to the center of sample.
(6) By using temperature–deformation cycles, the mutual ferrite/austenite phase ratio in the *x*-axis and *y*-axis directions changes in areas of high temperatures. In contrast, in the *z*-axis, this phase ratio remains unchanged. Thus, for a given temperature-stress state, the mutual ferrite/austenite ratio of about 62:38% can be considered as an equilibrium state. The applied temperature-stress cycle also affects the distribution of individual phases.
(7) The application of the temperature cycle with a maximum temperature of 1386 °C resulted in grain coarsening of ferrite and austenite in the areas of maximum temperatures. The degree of grain coarsening varied depending on the specific phase and direction (*x*, *y*, and *z*-axis).

## Figures and Tables

**Table 1:** Chemical composition (wt.%) of the X2CrMnNiN21-5-1 steel.

		C	Cr	Mn	Ni	Si
EN 10088-1	min.	-	21.00	4.00	1.35	-
	max.	<0.04	22.00	6.00	1.70	1.00
Experiment		0.039	21.45	4.86	1.51	0.72
		Mo	N	P	S	Cu
EN 10088-1	min.	0.10	0.20	-	-	0.10
	max.	0.80	0.25	0.04	0.015	0.80
Experiment		0.27	0.25	0.035	0.002	0.35

**Table 2:** Tensile test results for the basic material both in the rolling direction (RD) and in the direction perpendicular to the rolling direction (PD).

Strain Rate (s^−1^)	Sample No.	Sample Diameter (mm)	YS (MPa)	UTS (MPa)	A_g_ (%)	A_30_ (%)
10-2	RD-T23	6.50	703 ± 13	848 ± 1.5	16.53 ± 0.34	33.11 ± 1.06
10-2	PD-T23	6.50	687 ± 11	831 ± 3	17.24 ± 0.41	33.39 ± 1.29
	PD-T550	6.50	512 ± 7	756 ± 4	23.54 ± 0.51	37.68 ± 0.95
	PD-T650	6.50	482 ± 2	738 ± 3	29.51 ± 0.54	39.89 ± 2.37

**Table 3:** Percentage ratios of the individual phases along with the given directions: X, Y, and *z*-axis.

	*x*-axis		*y*-axis		*z*-axis	
	Basic Material	After Temperature Cycle of 1386 °C	Basic Material	After Temperature Cycle of 1386 °C	Basic Material	After Temperature Cycle of 1386 °C
Ratio of austenite	45.9	35.4	48.4	42.3	38.3	38.8
Ratio of ferrite	48.7	61.9	47.0	56.5	58.6	60.1
Unidentified	5.4	2.7	4.6	1.2	3.1	1.1

**Table 4:** Grain size of ferrite and austenite in the basic material and in the area achieving the maximal temperature of 1386 °C.

	*x*-axis		*y*-axis		*z*-axis	
	Basic Material	After Temperature Cycle of 1386 °C	Basic Material	After Temperature Cycle of 1386 °C	Basic Material	After Temperature Cycle of 1386 °C
Grain size of ferrite (µm)	13.02	17.89	11.80	14.38	20.45	21.62
Grain size of austenite (µm)	8.19	9.86	7.82	8.25	9.29	10.62

## Data Availability

Not applicable.
